# M. Falcony’s Powder for Preserving Dead Bodies

**Published:** 1859-01

**Authors:** 


M. FALCONY'S POWDER FOR PRESERVING DEAD BODIES.—The result of a very successful trial of M. Falcony's mode of preserving dead bodies was seen at the Grosvenor-place School on Tuesday. A body was brought to the school on the 24th of September in a state of decomposition so advanced as to be quite unfit for dissection. It was covered with M. Falcony's powder, and left in an open coffin until Tuesday last, when it was inspected in the presence of a number of scientific and literary men. There had not been the least offensive odor in the room in which the coffin was kept, and the body on Tuesday was quite free from odor. The powder used contains a large proportion of dried sulphate of zinc; this is mixed with common sawdust of white pine before covering the body. The rationale of the process is easily understood. The sawdust keeps the oxygen of the atmosphere from access to the body, and the emanations from the body are oxydized in the sawdust by the atmospheric oxygen. Hence there is no escape of the fetid gases. Then internal decomposition is prevented by the sulphate of zinc absorbing the water of the body, deliquescing, and recrystallizing as hydrate —probably with seven equivalents of water of crystallization. Whether this explanation be correct or not, there can be no doubt whatever that the process is a cheap, safe, and effectual one, perfectly fulfilling its intended object; and we feel sure that the adoption of such a process with all our dead, would tend to protect the living from cadaveric poisoning of the air we breathe and the water we drink.—*Med. Times and Gazette*, Oct. 23d, 1858.

